# Exploratory research on therapeutic agents combined with early diagnostic biomarkers for colorectal cancer

**DOI:** 10.3389/fphar.2025.1723072

**Published:** 2026-01-08

**Authors:** Qiming Liao, Yulong Xue, Yajiang Yu, Mengzhe Liu, Zeyuan Yu, Xiaoxia Cheng, Lei Zhang, Xinying Ji

**Affiliations:** 1 Department of Medical Informatics and Computer, Zhengzhou Health College, Zhengzhou, Henan, China; 2 School of Basic Medical Sciences, Henan University, Kaifeng, Henan, China

**Keywords:** colorectal cancer, differentially expressed genes, drug screening, molecular docking, biomarkers

## Abstract

**Introduction:**

Colorectal cancer (CRC) remains a leading cause of cancer-related mortality worldwide. Although diagnostic and therapeutic strategies have advanced, the molecular mechanisms driving CRC pathogenesis are not fully understood, highlighting the need for novel biomarkers and therapeutic agents.

**Methods:**

Integrated bioinformatics analyses of transcriptomic datasets from The Cancer Genome Atlas (TCGA) and Gene Expression Omnibus (GEO) were performed to identify survival-associated differentially expressed genes (DEGs) in CRC (|log2FC| > 1.5, p < 0.05). These key DEGs were then used to query the L1000FWD, DGIdb, and CMap platforms to predict candidate small-molecule drugs. The top candidate was evaluated by molecular docking, and its anti-tumor effects were validated by MTT cell viability assays in CRC cell lines.

**Results:**

We identified 15 survival-associated DEGs—MELK, NFE2L3, MCM2, MAD2L1, AUNIP, CXCL3, GLDN, GREM2, ALDH1A1, CILP, FABP4, AOC3, CNN1, ANGPTL1, and DES—as potential early diagnostic biomarkers for CRC. Drug-repositioning analyses convergently highlighted SB-225002 as a promising therapeutic agent. Molecular docking showed high binding affinity of SB-225002 to multiple key targets. MTT assays demonstrated that SB-225002 exerted dose-dependent inhibitory effects on the proliferation of CRC cell lines (SW-480, DLD-1, and MC38), with IC50 values of 2.307 μM, 0.9456 μM, and 3.449 μM, respectively.

**Discussion:**

This study systematically delineates a novel panel of early-detection biomarkers for CRC and identifies SB-225002 as a repurposed candidate therapeutic agent. The integrative strategy combining multi-cohort transcriptomic analysis, drug-repositioning platforms, molecular docking, and experimental validation offers a feasible framework for discovering clinically actionable biomarkers and small-molecule therapies for CRC.

## 1 Introduction

According to the 2022 Global Cancer Statistic report, CRC is the second leading cause of cancer-related deaths, accounting for 9.6% of newly diagnosed cancer cases and 9.3% of cancer-related deaths worldwide each year (Bray et al., 2024). Epidemiological research has shown that the incidence and mortality of CRC are closely associated with a spectrum of risk factors such as genetic variations, ethnicity, family history, age, high-fat intake, red meat consumption, obesity, alcohol consumption, smoking, and lack of physical activity (Shams-White et al., 2019). Currently, surgery and chemotherapy are the primary clinical treatment strategies for CRC (Salibasic et al., 2019). While Surgical treatment is mainly applicable to patients diagnosed at an early stage, approximately 60%–70% of symptomatic CRC cases are diagnosed at a later stage (Shinagawa et al., 2018). Despite aggressive clinical treatment regimens, the prognosis of patients with advanced CRC remains poor due to metastasis (Kvietkauskas et al., 2021). Therefore, there is an urgent need to identify effective pharmacological agents and develop novel therapeutic strategies to improve CRC prognosis.

With the rapid advancement of genomics, new opportunities have emerged for CRC treatment. Genomic technologies enable an in-depth understanding of the molecular pathogenesis of CRC and the identification of biomarkers and therapeutic targets associated with CRC prognosis (Van Schaeybroeck et al., 2011; Jiang et al., 2022). Drug repurposing has emerged as a highly promising strategy in oncological research, offering significant advantages over traditional drug development, including shorter development cycles, reduced costs, and a more favorable safety profile (Xia et al., 2024). This approach is particularly relevant for CRC, where recent biomarker studies have identified key therapeutic targets. For instance, Squalene Epoxidase (SQLE) has been found to promote CRC progression by inhibiting cancer cell apoptosis and disrupting the intestinal flora balance. Consequently, the conventional antifungal SQLE inhibitor, terbinafine, is now considered a potential therapeutic agent for CRC (Li et al., 2022). Similarly, the overexpression of Mitogen-Activated Protein Kinase Kinase 3 (MKK3) is known to drive CRC development. AT9283, a drug previously investigated for hematological malignancies, has been identified as a potential MKK3 inhibitor for CRC treatment (Piastra et al., 2024). Furthermore, recent studies have indicated that the traditional antipsychotic drug, aripiprazole, also exhibits anti-tumor efficacy against CRC (Hu et al., 2024). However, although numerous studies have identified DEGs in CRC, a significant challenge remains in systematically integrating these gene signatures with multi-omics drug databases to efficiently discover drug repurposing candidates for CRC therapy. Our study aims to bridge this gap. To this end, we utilized an integrated bioinformatics approach combining several powerful databases. Complementary to each other, these platforms offer distinct yet synergistic functionalities: the L1000 Fireworks Display (L1000FWD) platform visualizes drug-induced gene expression signatures, facilitating drug mechanism elucidation and repurposing (Wang et al., 2018). The Drug-Gene Interaction Database (DGIdb) provides comprehensive information on druggable genes and their interactions (Griffith et al., 2013; Freshour et al., 2021). The Connectivity Map (CMap) compares disease-associated gene expression profiles with those induced by small molecules, generating connectivity scores to identify potential therapeutics (Zhao et al., 2023). The synergy of these tools offers a novel methodology for linking CRC-associated gene signatures to potential drugs.

This study aims to identify potential candidate drugs for CRC treatment through a comprehensive analysis of gene expression data. We obtained CRC gene datasets from the GEO and TCGA databases and identified DEGs significantly associated with patient survival. These prognostic DEGs were subsequently used as inputs for simultaneous screening in the L1000FWD, DGIdb, and CMap databases to pinpoint small-molecule drugs with inverse correlation patterns. From the resulting candidates, SB-225002 was selected for further investigation. Molecular docking studies confirmed its strong binding affinity to multiple target proteins, and *in vitro* experiments demonstrated its potent anti-proliferative effects on CRC cell lines.

## 2 Materials and methods

### 2.1 Data acquisition and processing

Gene expression FPKM (Fragments Per Kilobase of transcript per Million mapped reads) values and clinical characteristic data of CRC samples were obtained from The Cancer Genome Atlas (TCGA) database. TCGA database offers the advantages of a large sample size, complete clinical annotations, and standardized quality control. Additionally, three independent CRC gene expression datasets (GSE18105 (Matsuyama et al., 2010), GSE20842 (Gaedcke et al., 2010), and GSE87211 (Hu et al., 2018)) containing tumor and normal tissue samples were obtained from the Gene Expression Omnibus (GEO) database (Table 1). Microarray data were processed using the limma package for background correction and quantile normalization. Batch effects were then corrected using the ComBat algorithm from the sva package. Finally, genes with low expression across all samples were filtered out, and samples from the TCGA cohort with missing survival time or event status were excluded.

**TABLE 1:** Relevant information of each data set.

Data set	Number of samples used (number)	
	Normal	Tumors
TCGA-CRC	44	620
GSE18105	17	94
GSE20842	65	65
GSE87211	160	203

### 2.2 Clinical sample collection

Between December 2023 and April 2024, four fresh tumor tissue samples (along with paired normal tissue samples) were collected from CRC patients who underwent surgery at Huaihe Hospital of Henan University, China. All samples were stored at −80 °C for preservation. Prior to surgery, all patients were fully informed about the study and signed an informed consent form. This study was reviewed and approved by the Clinical Research Ethics Committee of Huaihe Hospital of Henan University. Ethical Approval Code: IEC-C-010-A07-V2.0.

### 2.3 Total RNA extraction

Approximately 0.5 cm × 0.5 cm of tissue was collected and homogenized in 200 μL of Trizol lysis buffer using a tissue homogenizer. An additional 800 μL of Trizol was added, and the mixture was incubated at room temperature for 5 min. Subsequently, 200 μL of chloroform was added, followed by thorough mixing by inversion for 3 min. The samples were then centrifuged at 12,000 × g for 15 min at 4 °C, and the aqueous phase was transferred to a new RNase-free EP tube. An equal volume of isopropanol was added, mixed, and incubated at room temperature for 10 min. The mixture was centrifuged again at 12,000 × g for 10 min at 4 °C, and the supernatant was discarded. The RNA pellet was washed with 1 mL of 75% ethanol, followed by centrifugation at 7,500 × g for 5 min at 4 °C. After discarding the supernatant, the RNA pellet was air-dried for 5–10 min, then resuspended in RNase-free water (Chomczynski and Sacchi, 2006).

### 2.4 RNA sequencing

Extracted total RNA was sent to a sequencing company for transcriptome sequencing (RNA-seq). RNA sequencing libraries were constructed, and sequencing was performed using the Illumina NovaSeq 6000 platform (PE150 mode) with a sequencing depth of 10 Gb reads per sample.

### 2.5 Differential gene expression analysis of CRC

The limma package in *R* was used to analyze DEGs between CRC tissues and normal tissues from public datasets (TCGA-CRC, GSE18105, GSE20842, and GSE87211) and our collected clinical samples. Genes with *p* < 0.05 and |log_2_ FC| > 1.5 were considered significantly differentially expressed.

### 2.6 Differential gene expression and functional enrichment analysis

Diffrentially Expressed Genes (DEGs)were identified using the “limma” R package, with a threshold of adjusted *p* < 0.05 and |log_2_ FC| > 1.5. The overlapping significantly up- and downregulated differentially expressed genes (DEGs) common to both the TCGA and GEO datasets were identified. To elucidate their biological functions, Gene Ontology (GO) and Kyoto Encyclopedia of Genes and Genomes (KEGG) pathway enrichment analyses were subsequently performed using the “clusterProfiler” R package. Visualization of the enrichment results was conducted using the “ggplot2” R package.

### 2.7 Screening of prognosis-associated genes

To investigate the impact of DEGs on CRC prognosis, univariate Cox regression analysis was performed using “survival” R packages. Genes with a significant association with CRC prognosis were identified using univariable Cox proportional hazards regression (*p* < 0.05), and Kaplan–Meier survival curves along with log-rank tests were used to compare survival differences between high- and low-expression groups.

### 2.8 Validation of gene expression

To enhance the reliability of the identified DEGs, validation was performed using our own sequencing data. Genes that |log_2_ FC| > 1.5 and *p* < 0.05 in differential expression analysis, as well as *p* < 0.05 in t-tests, were included in the subsequent drug prediction analysis.

The Human Protein Atlas (HPA) online database (www.proteinatlas.org) provides information on the tissue and cellular distribution of 26,000 human proteins, primarily using specific antibodies to analyze protein expression in cell lines, normal tissues, and tumor tissues (Gu et al., 2020). We utilized this database to verify the expression patterns of 15 key DEGs in normal colon tissue and CRC tissue.

### 2.9 Screening of small-molecule drugs

L1000FWD (https://maayanlab.cloud/l1000fwd/), DGIdb (https://www.dgidb.org/), and CMap (https://clue.io/) are essential tools for drug discovery research (Wen and Ablimit, 2024; Cannon et al., 2024; Gao et al., 2019). We analyzed CRC-associated DEGs that were significantly related to patient survival using these three databases. Small-molecule drugs that exhibited an inverse correlation with these DEGs were identified, leading to the determination of potential candidate drugs for CRC treatment.

### 2.10 Molecular docking

PubChem is the world’s largest public database for chemical information (Kim et al., 2025), while Universal Protein (UniProt) is one of the most comprehensive and widely used protein databases, containing over 220 million protein entries (Wang et al., 2021). The chemical data and structures of the selected drugs were retrieved from PubChem (https://pubchem.ncbi.nlm.nih.gov/), and the X-ray crystal structures of the target proteins were obtained from UniProt (https://www.uniprot.org/). Only human protein structures were used.

To evaluate the screened small-molecule drugs, molecular docking was performed to examine their binding reliability to target proteins based on binding energy calculations. Molecular docking studies were conducted using SYBYL-X software (Palafox et al., 2023). The Surflex-Dock module in SYBYL-X 2.1.1 was used for protein preprocessing, including removal of crystallographic water and ligands, addition of hydrogen atoms and charges, and force field optimization. The “automatic” mode was used to generate active binding sites on the target protein, followed by docking with small-molecule drug Mol2 files. The docking results were visualized using PyMOL 2.6, a widely used software for the visualization and analysis of biomolecules (Rosignoli and Paiardini, 2022).

### 2.11 MTT assay

The MTT assay is widely used to assess the cytotoxic effects of drugs on cells by evaluating cell viability (Ghasemi et al., 2023). CRC cells in the logarithmic growth phase were digested, centrifuged, and counted, then seeded in 96-well plates. For drug treatment, the selected drug (SB-225002) was prepared into a series of gradient concentrations (0.0075, 0.195, 0.39, 1.56, 3.125, 6.25, 12.5, 25 μM) using complete medium; 100 μL of the drug solution, with three replicate wells per concentration. After that, cells were incubated in a 37 °C, 5% CO_2_ cell culture incubator (Thermo Scientific, United States) for 48 h. Subsequently, 15 μL of MTT solution (5 mg/mL) was added to each well, followed by incubation at 37 °C for 4 h under light-protected conditions. The medium and MTT were discarded, and 100 μL of dimethyl sulfoxide (DMSO) was added. Optical density (OD) was measured at 490 nm using a microplate reader. The IC_50_ value was calculated using GraphPad software.

### 2.12 Statistical analysis

Statistical analyses were conducted using GraphPad Prism 8.0 and R software (version 4.2.2). All experimental data are presented as mean ± standard deviation (SD). Continuous variables with a normal distribution were analyzed using t-tests. For data not following a normal distribution, log_2_ transformation was applied prior to t-test analysis. Kaplan–Meier survival analysis was performed using the log-rank test. *p* < 0.05 was considered statistically significant.

## 3 Results

### 3.1 Identification of differentially expressed genes

**FIGURE 1:**
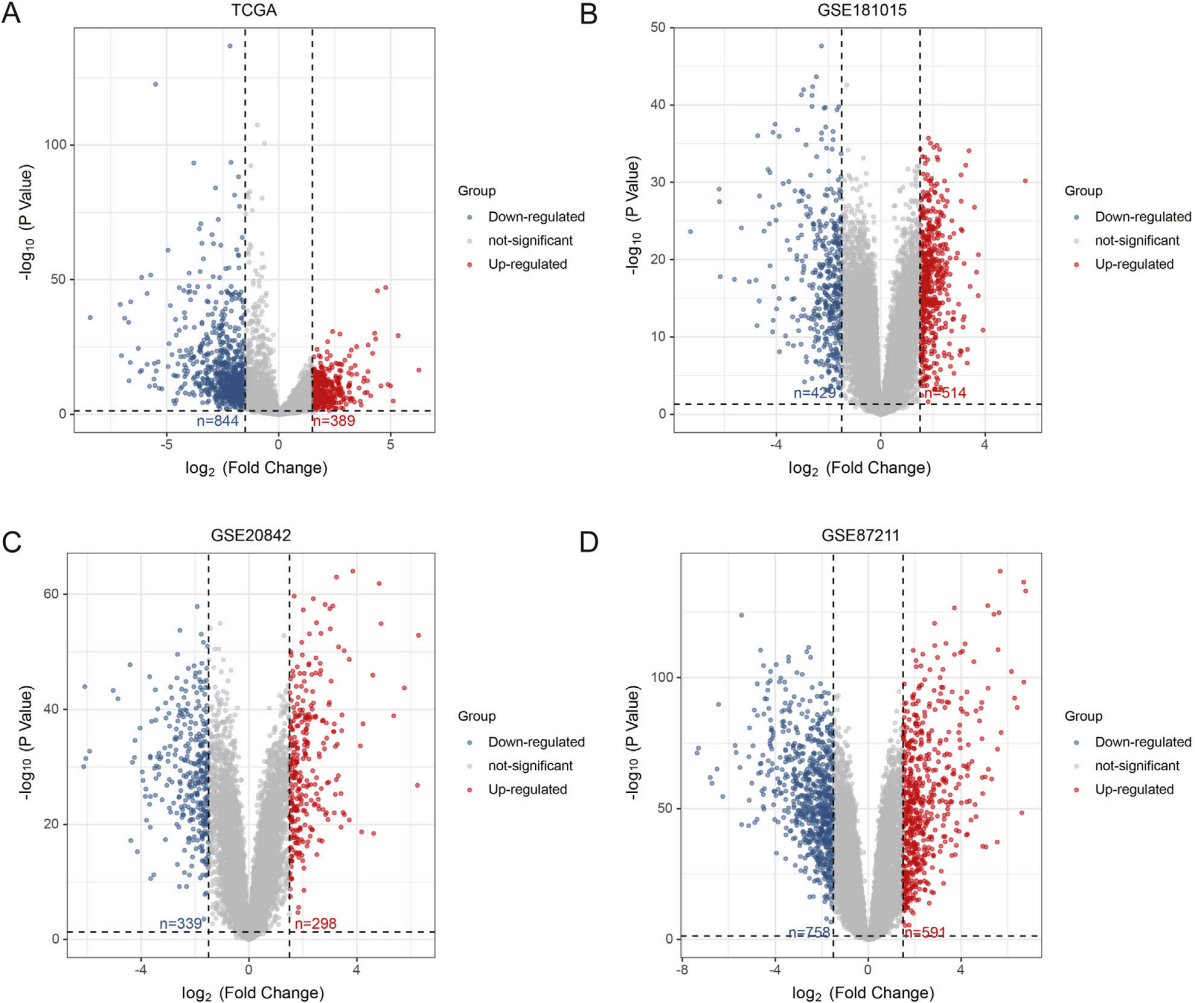
Differential Expression Analysis Based on GEO and TCGA Databases. **(A)** TCGA-CRC, **(B)** GSE18105, **(C)** GSE20842, **(D)** GSE87211. The red dots represent the upregulated genes based on an adjusted P < 0.05 and |log fold change| > 1.5; the blue dots represent the downregulated genes based on an adjusted P < 0.05 and |log fold change| > 1.5; the black spots represent genes with no significant difference in expression.

**FIGURE 2:**
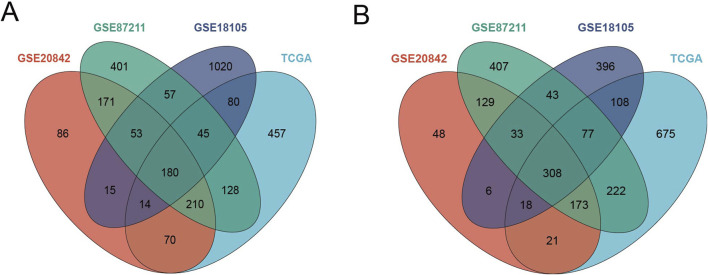
Intersection Analysis of Differentially Expressed Genes. **(A)** Venn Diagram of Upregulated Genes: Intersection of upregulated genes from the TCGA-CRC, GSE18105, GSE20842, and GSE87211 datasets. **(B)** Venn Diagram of Downregulated Genes: Intersection of downregulated genes from the same datasets.

In the TCGA-CRC dataset, a total of 1,233 DEGs were identified, including 389 upregulated genes and 844 downregulated genes. After normalization, differential expression analysis was performed on the CRC microarray datasets GSE18105, GSE20842, and GSE87211. Specifically, the GSE18105 dataset contained 944 DEGs, with 515 upregulated and 429 downregulated genes. The GSE20842 dataset had 637 DEGs, including 298 upregulated and 339 downregulated genes. The GSE87211 dataset identified 1,349 DEGs, with 591 upregulated and 758 downregulated genes. The DEGs from these four datasets are presented in Figure 1. A Venn diagram analysis of upregulated and downregulated genes across the datasets identified 180 common upregulated genes (Figure 2A) and 308 common downregulated genes (Figure 2B).

### 3.2 KEGG and GO enrichment analysis of DEGs

**FIGURE 3:**
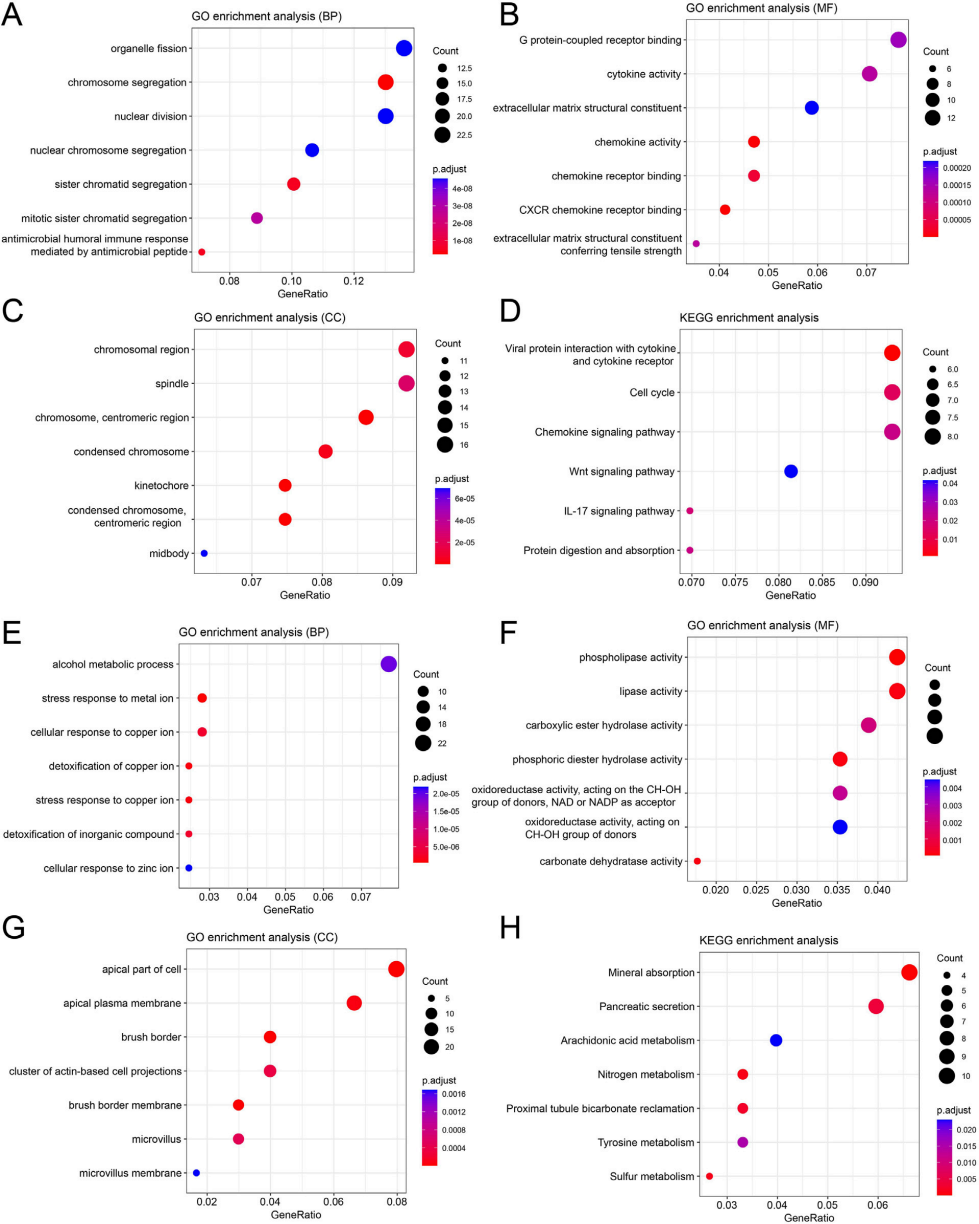
Enrichment analysis of differentially expressed genes. **(A–D)** enrichment of upregulated genes: **(A)** Biological process; **(B)** molecular function; **(C)** cellular component; **(D)** KEGG pathway; **(E–H)** enrichment of downregulated genes; **(E)** biological process; **(F)** molecular function; **(G)** cellular component; **(H)** KEGG pathway.

GO and KEGG enrichment analyses were conducted for the common DEGs. The biological process (BP) enrichment analysis of upregulated genes revealed that these genes were significantly involved in organelle fusion, chromosome segregation, and nuclear division (Figure 3A). Molecular function (MF) enrichment analysis indicated strong associations with G-protein coupled receptor binding, cytokine activity, and chemokine receptor binding (Figure 3B). Cellular component (CC) enrichment analysis showed significant enrichment in chromosome regions, spindle apparatus, and centromeric regions (Figure 3C). KEGG pathway enrichment analysis suggested that these genes were likely involved in viral protein interactions with cytokines, the cell cycle, and the chemokine signaling pathway (Figure 3D). For the downregulated genes, BP enrichment analysis indicated associations with alcohol metabolism, stress response to metal ions, and copper detoxification (Figure 3E). MF enrichment analysis demonstrated significant relationships with phospholipase activity, lipase activity, and carboxylesterase hydrolase activity (Figure 3F). CC enrichment analysis showed enrichment in apical parts of the plasma membrane, brush border, and microvilli (Figure 3G). KEGG pathway analysis suggested that these genes were involved in mineral absorption, arachidonic acid metabolism, and nitrogen metabolism (Figure 3H). These results provide crucial insights into the biological roles of the identified DEGs.

### 3.3 Screening of therapeutic targets

**FIGURE 4:**
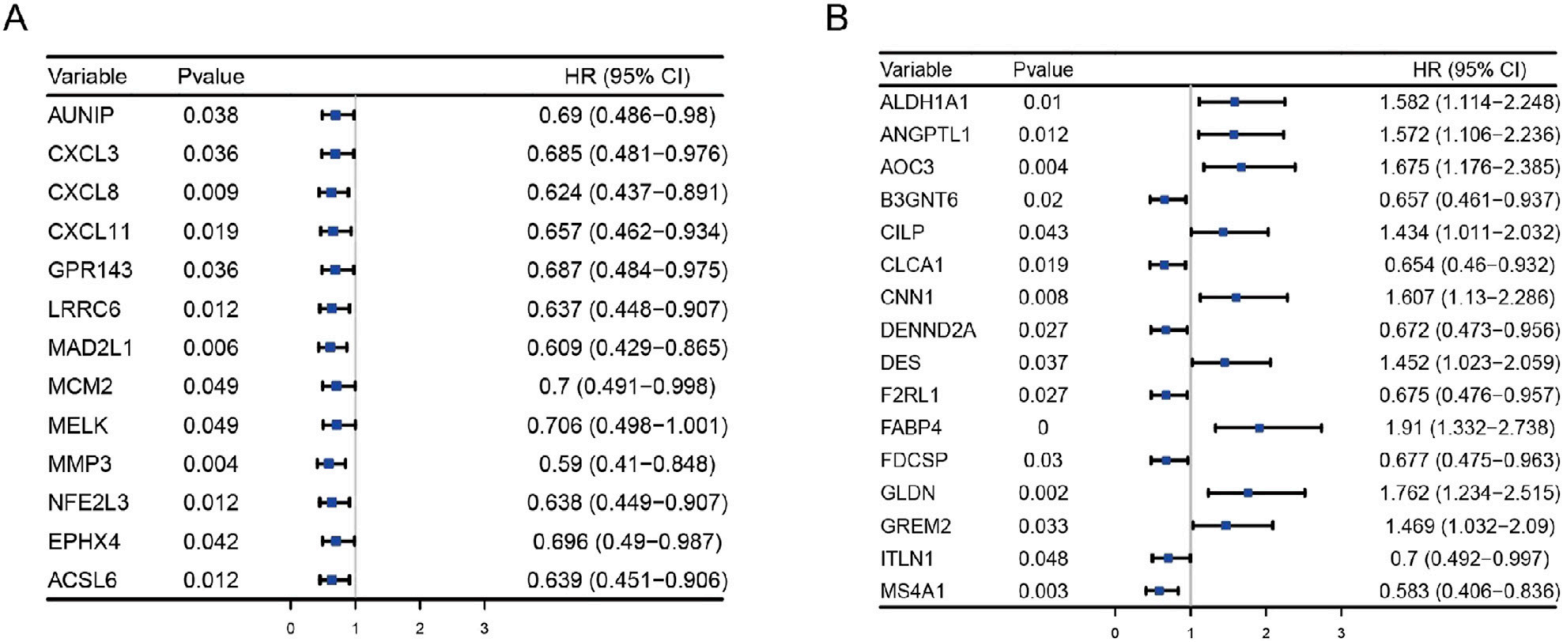
Univariate COX Regression Analysis of Gene Prognostic Relevance. **(A)** Prognostic significance of 13 upregulated genes; **(B)** Prognostic significance of 16 downregulated genes.

**FIGURE 5:**
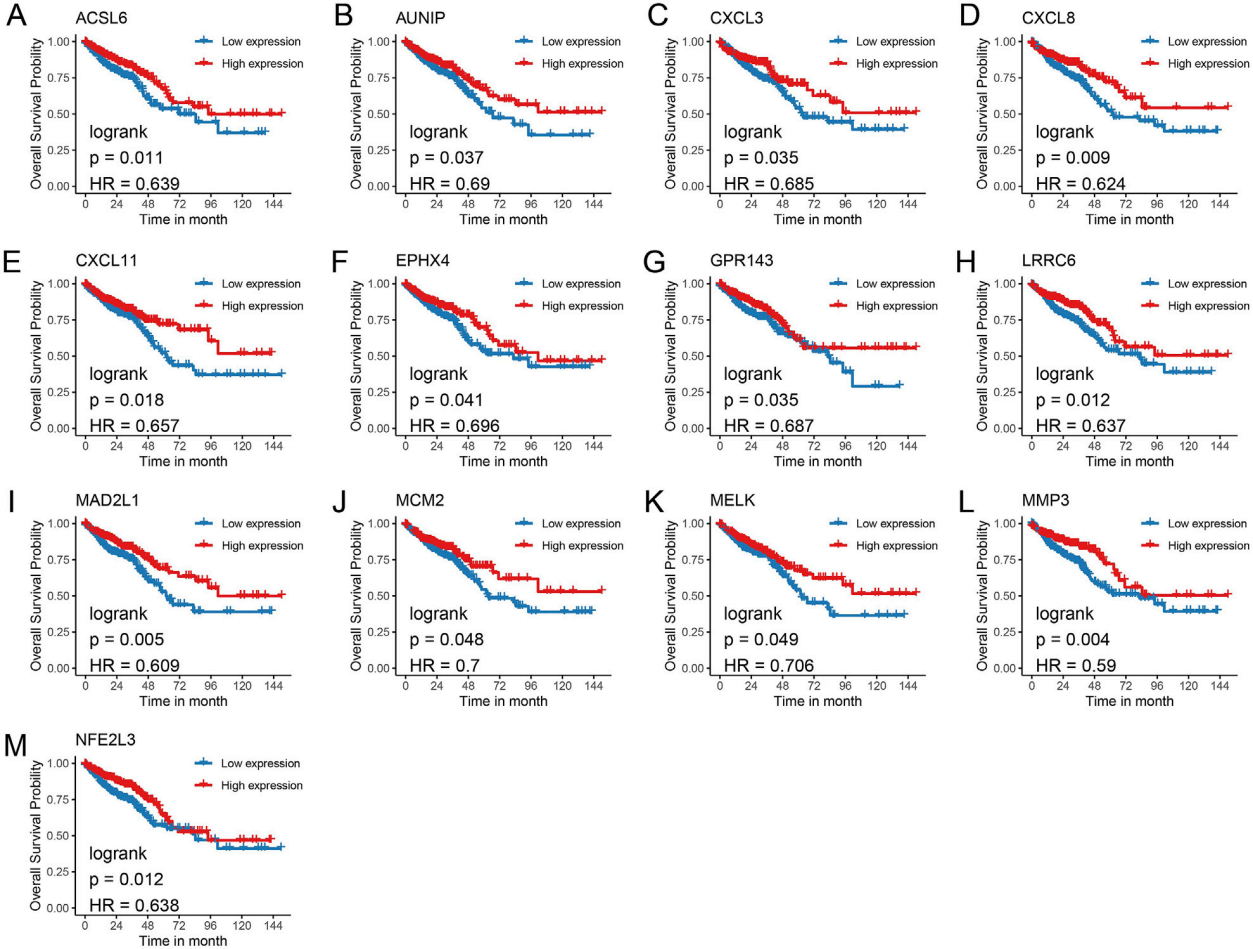
Kaplan-Meier survival curves for genes with upregulated expression. **(A)** ACSL6; **(B)** AUNIP; **(C)** CXCL3; **(D)** CXCL8; **(E)** CXCL11; **(F)** EPHX4; **(G)** GPR143; **(H)** LRRC8; **(I)** MAD2L1; **(J)** MCM2; **(K)** MELK; **(L)** MMP3; **(M)** NFE2L3.

**FIGURE 6:**
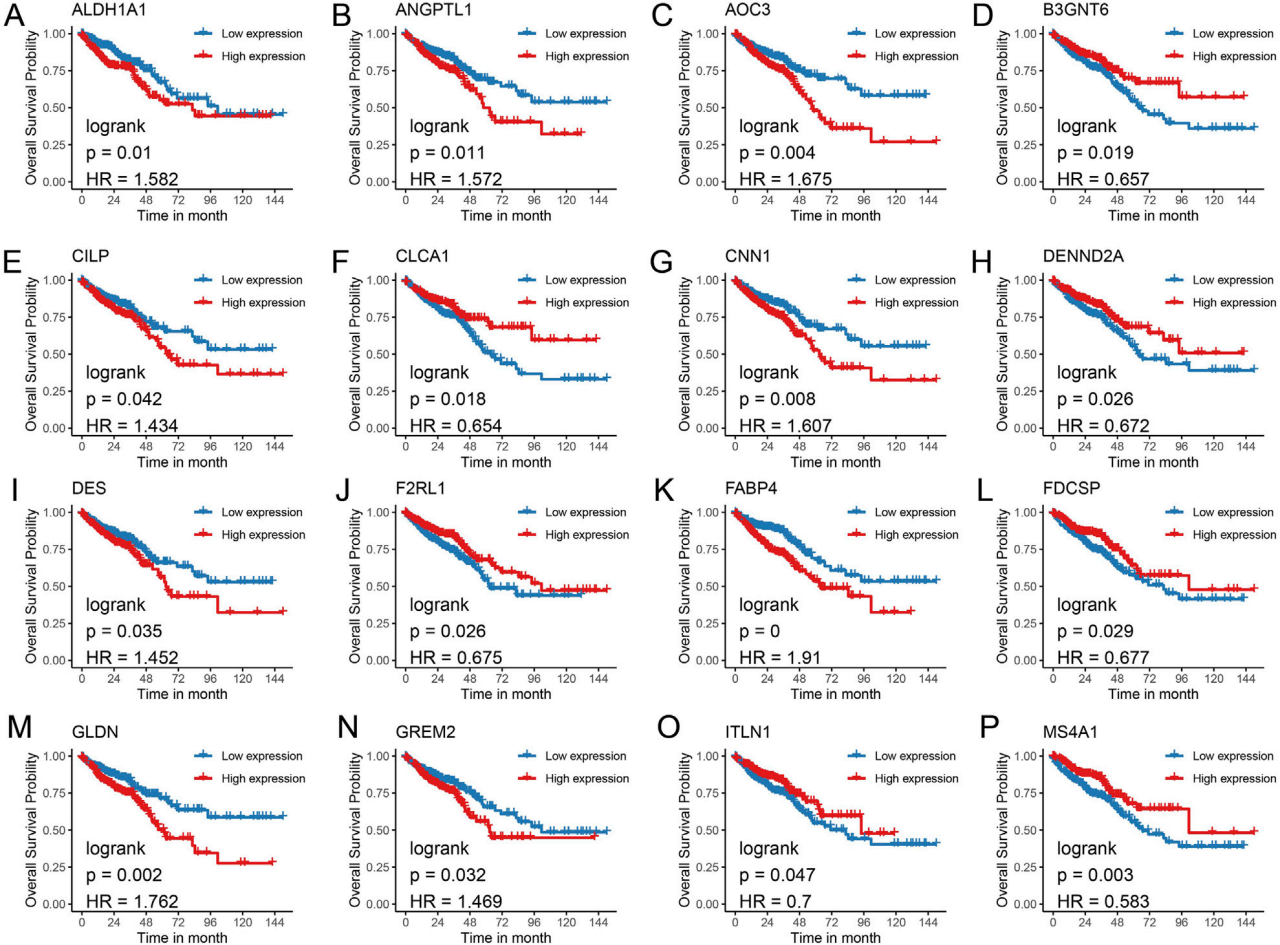
Kaplan-Meier survival curves for genes with downregulated expression. **(A)** ALDH1A1; **(B)** ANGPTL1; **(C)** AOC3; **(D)** B3GNT6; **(E)** CLP; **(F)** CLCA1; **(G)** CNN1; **(H)** DENND2A; **(I)** DES; **(J)** F2RL1; **(K)** FABP4; **(L)** FDCSP; **(M)** GLDN; **(N)** GREM2; **(O)** ITLN1; **(P)** MS4A1.

To identify key genes with the most significant impact on CRC prognosis, a univariate Cox regression model was applied to the 180 commonly upregulated genes, revealing 13 genes (*ACSL6*, *AUNIP*, *CXCL3*, *CXCL8*, *CXCL11*, *EPHX4*, *GPR143*, *LRRC8*, *MAD2L1*, *MCM2*, *MELK*, *MMP3*, and *NFE2L3*) associated with overall survival (OS), all of which acted as protective factors (Figure 4A). Among the 308 commonly downregulated genes, 16 genes were significantly correlated with OS (Figure 4B), with 9 genes (*ALDH1A1*, *ANGPTL1*, *AOC3*, *CLP*, *CNN1*, *DES*, *FABP4*, *GLDN*, and *GREM2*) acting as risk factors, while seven genes (B3GNT6, *CLCA1*, *DENND2A*, *F2RL1*, *FDCSP*, *ITLN1*, and *MS4A1*) functioned as protective factors. Kaplan-Meier survival curves were generated for the 13 upregulated genes (Figure 5) and the 16 downregulated genes (Figure 6), demonstrating statistically significant differences (log-rank *p* < 0.05).

**FIGURE 7:**
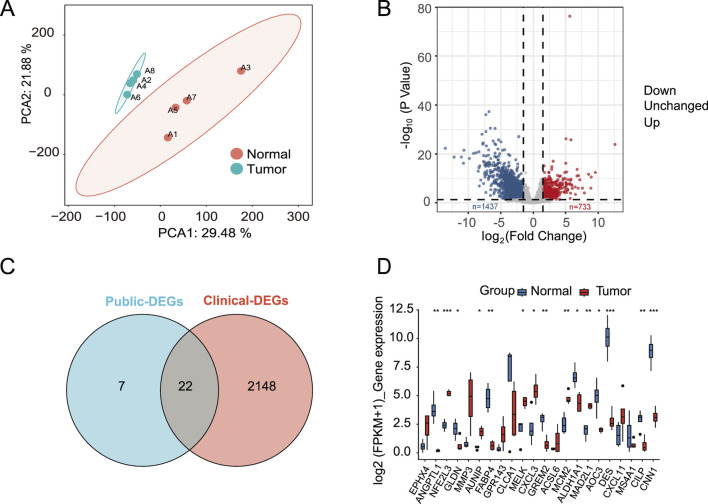
Transcriptome Analysis and Differential Gene Expression Verification in CRC Tissues and Normal Tissues. **(A)** Two-dimensional Principal Component Analysis (PCA) of transcriptome sequencing samples; **(B)** Differentially Expressed Genes (DEGs) between CRC tissues and normal tissues in clinical tumor samples; **(C,D)** Parametric tests for the expression levels of common differential genes identified in both public datasets and this study’s samples, *P < 0.05, **P < 0.01, ***P < 0.001.

Principal Component Analysis (PCA) was performed on clinical tumor samples to assess group differences and biological reproducibility within groups. As shown in (Figure 7A), samples within each group were highly similar, whereas CRC tissues and normal tissues exhibited significant inter-group differences, supporting further transcriptional profiling analyses. Differential expression analysis between CRC and normal tissues identified 2170 DEGs, with 1437 upregulated genes and 733 downregulated genes (Figure 7B). Intersecting these with 29 prognostic DEGs from four public datasets yielded 22 common genes (Figure 7C). To ensure robustness, we further validated these 22 candidates using a t-test on our clinical sequencing data (log_2_(FPKM+1)), which confirmed 15 significant DEGs (*p* < 0.05) for subsequent drug screening (Figure 7D).

### 3.4 Validation of DEGs at the protein level

**FIGURE 8:**
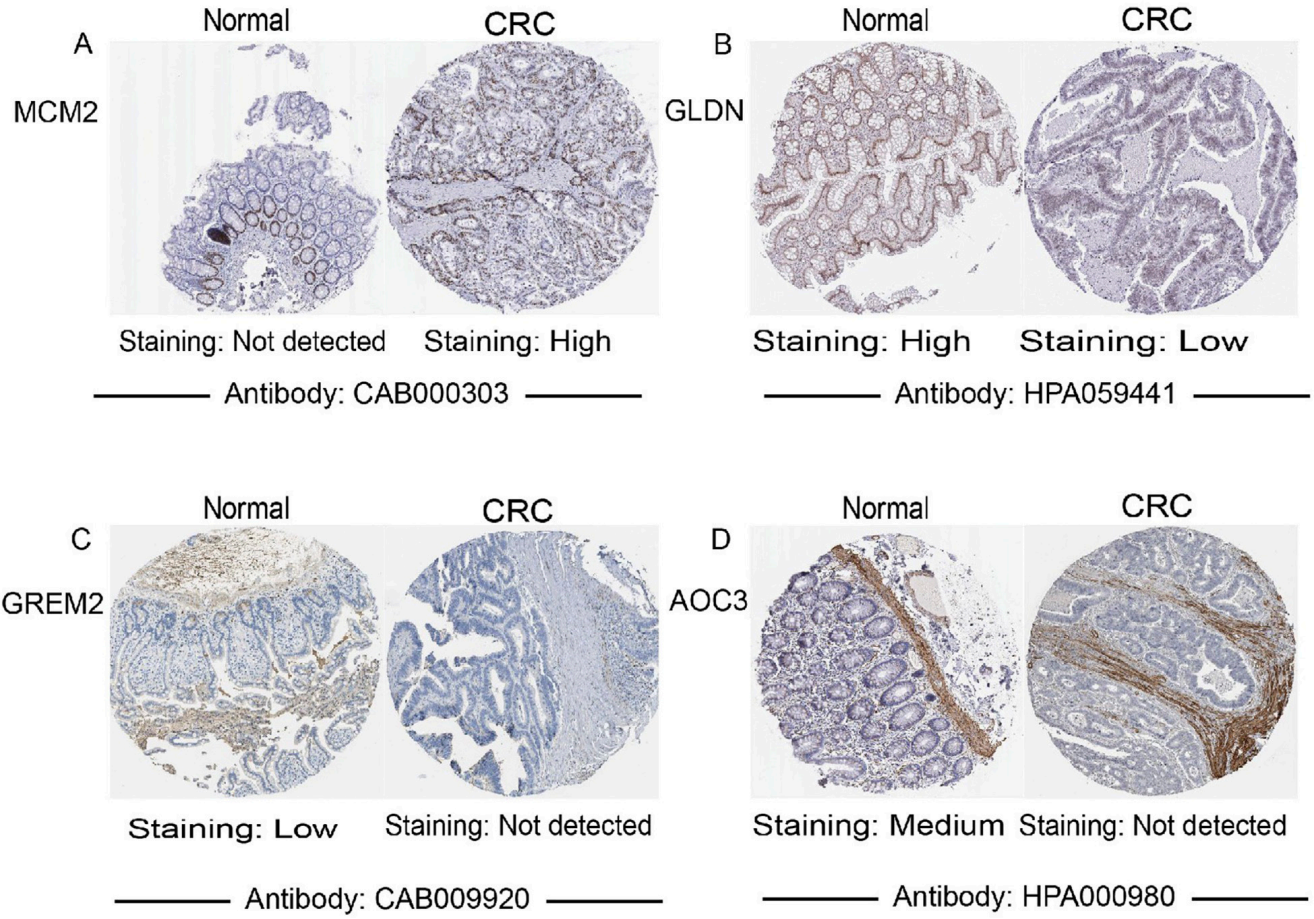
The protein expression levels of 15 key genes in normal intestinal tissue and CRC tissue were verified through the HPA online website. **(A)** MCM2; **(B)** GLDN; **(C)** GREM2; **(D)** AOC3. (MELK, NFE2L3, MAD2L1, AUNIP, CXCL3, ALDH1A1, CILP, FABP4, CNN1, ANGPTL1, DES were not found).

Protein expression levels of four key genes were analyzed using immunohistochemistry data from the Human Protein Atlas (HPA) database. Among them, CXCL3 expression was not detected. As shown in (Figure 8), compared with normal colonic tissues, MMP3 and NFE2L3 were upregulated in CRC tissues, while CLCA1 was downregulated, aligning with our previous predictions.

### 3.5 Online drug screening for candidate small molecules

**FIGURE 9:**
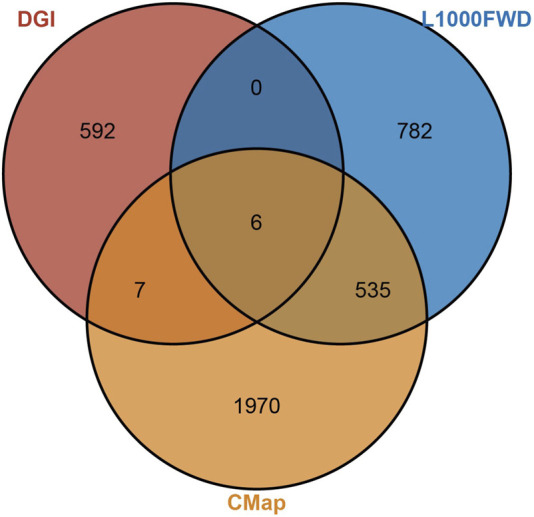
Intersection of potential therapeutic targets identified from the L1000FWD, DGIdb, and CMap databases.

Using the L1000FWD, DGIdb, and CMap databases, six upregulated genes and nine downregulated gene were used for a drug screening analysis, identifying six small-molecule drug candidates: MG-132, H-89, BMS-387032, JNJ-7706621, NVP-TAE684, and SB-225002 (Figure 9). MG-132, a proteasome inhibitor, showed anticancer effects but had severe side effects due to widespread inhibition of protein degradation (Fatima et al., 2020). H-89, a PKA inhibitor, was effective only in cancers driven by the PKA signaling pathway, limiting its broader application (Xu et al., 2015). BMS-387032 and JNJ-7706621, CDK kinase inhibitors, and NVP-TAE684, an ALK inhibitor, were too target-specific, restricting their clinical applicability (Ki et al., 2022). SB-225002, a chemokine receptor antagonist, modulates inflammatory cell migration, potentially inhibiting tumor-associated inflammation and disrupting pro-tumor inflammatory pathways, making it a promising candidate for cancer treatment (Sun and Yuan, 2022). Based on the screening results from the three platforms, and after a comprehensive evaluation of their mechanisms of action and potential side effects, SB-225002 was selected as the most promising candidate drug.

### 3.6 Molecular docking validation

**FIGURE 10:**
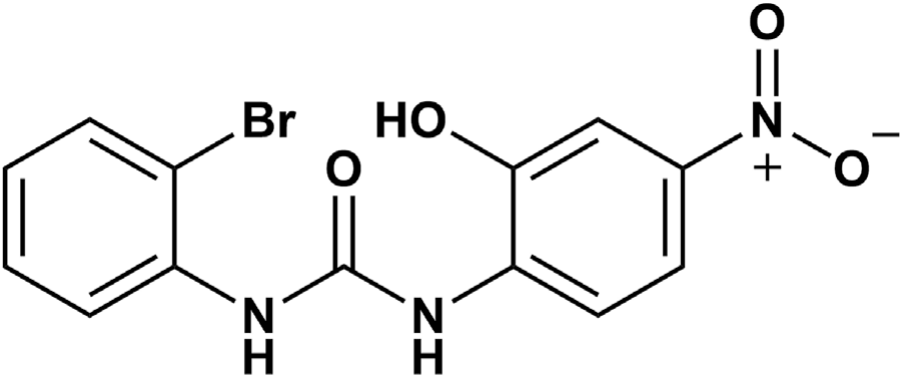
Two-dimensional structure of SB-225002.

Detailed information about SB-225002 was obtained from PubChem (https://pubchem.ncbi.nlm.nih.gov/). SB-225002 (PubChem CID: 3854666) has a molecular weight of 352.14 g/mol and a molecular formula of C_13_H_10_BrN_3_O_4_. The 2D structure of the compound is shown in (Figure 10). The SDF file of SB-225002 was downloaded from PubChem and converted into a Mol2 file using Open Babel GUI for use as a ligand in molecular docking studies.

The 3D structures of the target proteins were retrieved and prepared for docking, including MELK (PDB: 4IXP), NFE2L3 (AlphaFoldDB: AF-Q9Y4A8-F1-v4), MCM2 (AlphaFoldDB: AF-P49736-F1-v4), MAD2L1 (PDB: 3GMH), AUNIP (AlphaFoldDB: AF-Q9H7T9-F1-v4), CXCL3 (AlphaFoldDB: AF-P19876-F1-v4), GLDN (AlphaFoldDB: AF-Q6ZMI3-F1-v4), GREM2 (AlphaFoldDB: AF-Q9H772-F1-v4), ALDH1A1 (AlphaFoldDB: AF-P00352-F1-v4), CILP (AlphaFoldDB: AF-O75339-F1-v4), FABP4 (PDB: 3Q6L), AOC3 (PDB: 1PU4), CNN1 (AlphaFoldDB: AF-P51911-F1-v4), ANGPTL1 (AlphaFoldDB: AF-O95841-F1-v4), and DES (AlphaFoldDB: AF-P17661-F1-v4). These proteins were used as receptors for docking analysis.

High-precision molecular docking was performed using SYBYL-X 2.1.1. The docking scores included Total-score, CSCORE, Crash, and D-score. In this study, Total-score and CSCORE were used as the primary indicators of binding affinity. In general, a Total-score close to 4.0 indicates a reasonable binding activity, with higher scores suggesting stronger binding affinity (Feng et al., 2022). Similarly, a CSCORE approaching 4.0 signifies better spatial complementarity and energy compatibility between the ligand and receptor (Xue et al., 2023). The detailed docking results are presented in Table 2.

**TABLE 2:** Docking results of SB-225002 with potential therapeutic targets.

Target	Total score	D score	PMF score	Glide-score	CSCORE
MELK	4.7678	−91.2641	−42.4144	−146.1041	4
NFE2L3	2.8089	−47.2814	−25.5917	−59.5947	4
MCM2	3.24	−94.0047	−66.7782	−161.5184	5
MAD2L1	2.6392	−400.8736	−8.9056	−65.3208	4
AUNIP	4.7332	−79.4895	−5.9211	−129.0176	2
CXCL3	3.2541	−57.7457	−1.1214	−81.3206	4
GLDN	4.2143	−87.8545	−52.1828	−163.604	3
GREM2	4.263	−67.3437	−6.3226	−138.1529	4
ALDH1A1	3.5945	−105.8139	−24.9357	−152.1509	5
CILP	4.7971	−95.1384	−10.4182	−156.3967	2
FABP4	3.019	−116.4449	−42.1003	−177.1461	1
AOC3	4.8243	−103.4636	−72.2921	−168.5001	4
CNN1	3.6668	−88.0676	−20.1439	−122.1976	2
ANGPTL1	4.5888	−73.1425	−16.7597	−161.2542	4
DES	2.9105	−70.9133	−6.3249	−127.7536	2

**FIGURE 11:**
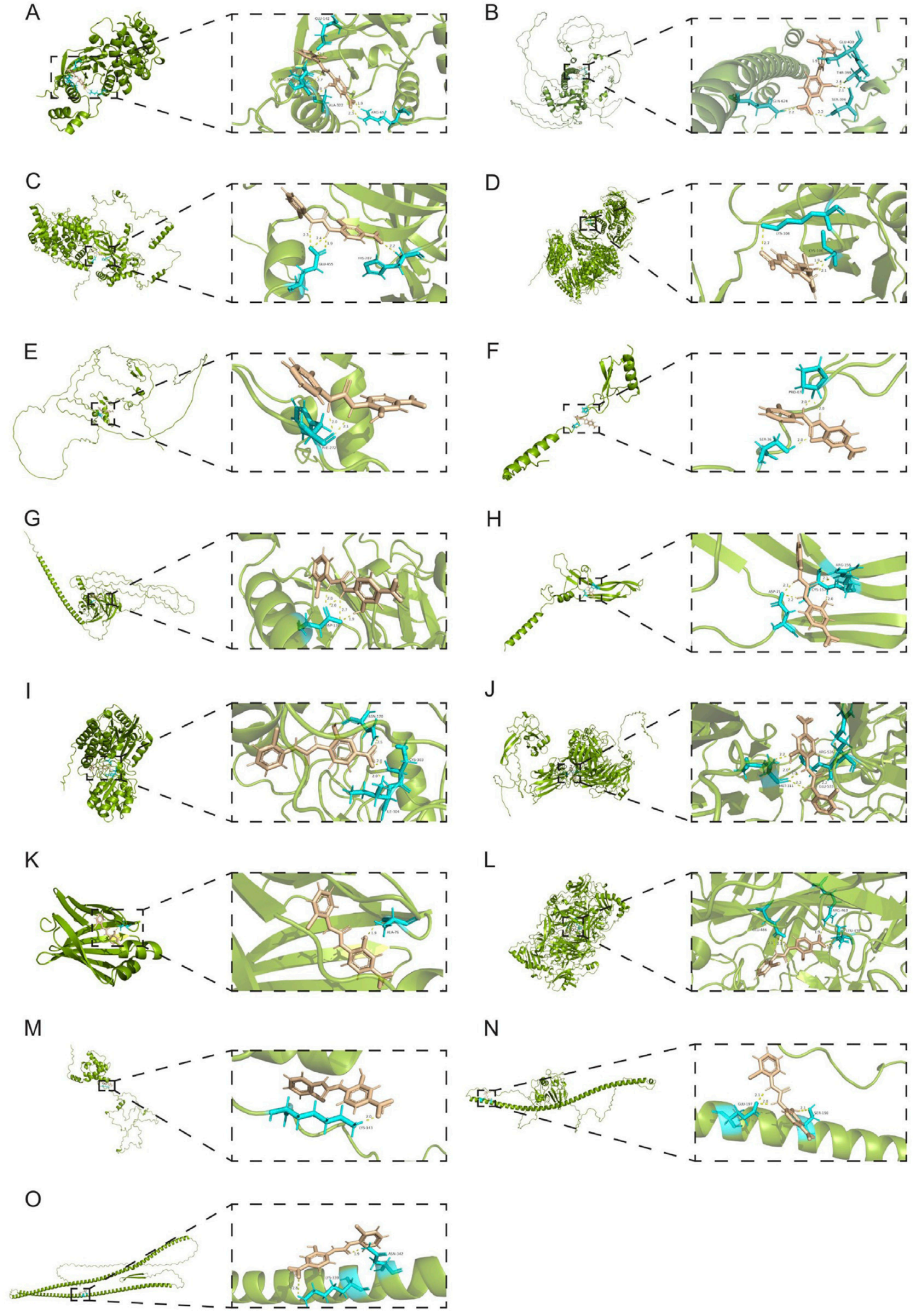
Molecular Docking verification of SB-225002 with the target protein. **(A)** MELK; **(B)** NFE2L3; **(C)** MCM2; **(D)** MAD2L1; **(E)** AUNIP; **(F)** CXCL3; **(G)** GLDN; **(H)** GREM2; **(I)** ALDH1A1; **(J)** CILP; **(K)** FABP4; **(L)** AOC3; **(M)** CNN1; **(N)** ANGPTL1; **(O)** DES. In this figure, green representatives the target protein; Brown indicates SB‐225002; The protein residues that interact with SB‐225002 are shown in blue. Yellow indicates hydrogen bonds.

SB-225002 formed five hydrogen bonds with MELK, specifically at ARG-65 (1.9 Å, 2.3 Å), ALA-322 (2.0 Å), GLU-142 (2.2 Å), and ARG-326 (2.7 Å) (Figure 11A). It also formed five hydrogen bonds with NFE2L3 at SER-396 (2.2 Å, 2.1 Å), GLN-624 (2.2 Å), THR-399 (2.4 Å), and GLU-400 (1.9 Å) (Figure 11B). For MCM2, SB-225002 established four hydrogen bonds at GLU-455 (1.9 Å, 2.3 Å, 2.4 Å) and HIS-287 (2.2 Å) (Figure 11C). With MAD2L1, it formed three hydrogen bonds at CYS-106 (1.9 Å, 2.1 Å) and LYS-108 (2.7 Å) (Figure 11D). For AUNIP, two hydrogen bonds were observed at PHE-272 (2.0 Å, 2.1 Å) (Figure 11E).

SB-225002 also interacted with CXCL3, forming three hydrogen bonds at SER-36 (2.0 Å) and PRO-67 (2.0 Å, 2.0 Å) (Figure 11F). With GLDN, it established four hydrogen bonds at ASP-110 (2.0 Å, 2.0 Å, 2.7 Å, 1.9 Å) (Figure 11G). For GREM2, four hydrogen bonds were observed at ASP-71 (2.1 Å, 2.2 Å), CYS-157 (2.0 Å), and ARG-156 (2.1 Å) (Figure 11H). The docking of SB-225002 with ALDH1A1 resulted in three hydrogen bonds at ILE-304 (2.0 Å), CYS-303 (2.0 Å), and ASN-170 (2.5 Å) (Figure 11I).

Further analysis revealed six hydrogen bonds between SB-225002 and CILP at MET-311 (1.9 Å, 2.0 Å, 2.2 Å, 2.3 Å), ARG-536 (2.0 Å), and GLU-535 (2.2 Å) (Figure 11J). Additionally, one hydrogen bond was formed with CILP at ALA-76 (1.9 Å) (Figure 11K). The docking with AOC3 resulted in four hydrogen bonds at GLU-486 (2.1 Å, 2.5 Å), ARG-463 (1.9 Å), and LEU-438 (2.0 Å) (Figure 11L). CNN1 formed one hydrogen bond with SB-225002 at LYS-143 (2.0 Å) (Figure 11M). ANGPTL1 established three hydrogen bonds at GLU-197 (2.0 Å, 2.1 Å) and SER-190 (2.1 Å) (Figure 11N). Finally, DES formed two hydrogen bonds at LYS-339 (2.8 Å) and ASN-342 (1.9 Å) (Figure 11O).

### 3.7 *In vitro* experimental validation

**FIGURE 12:**
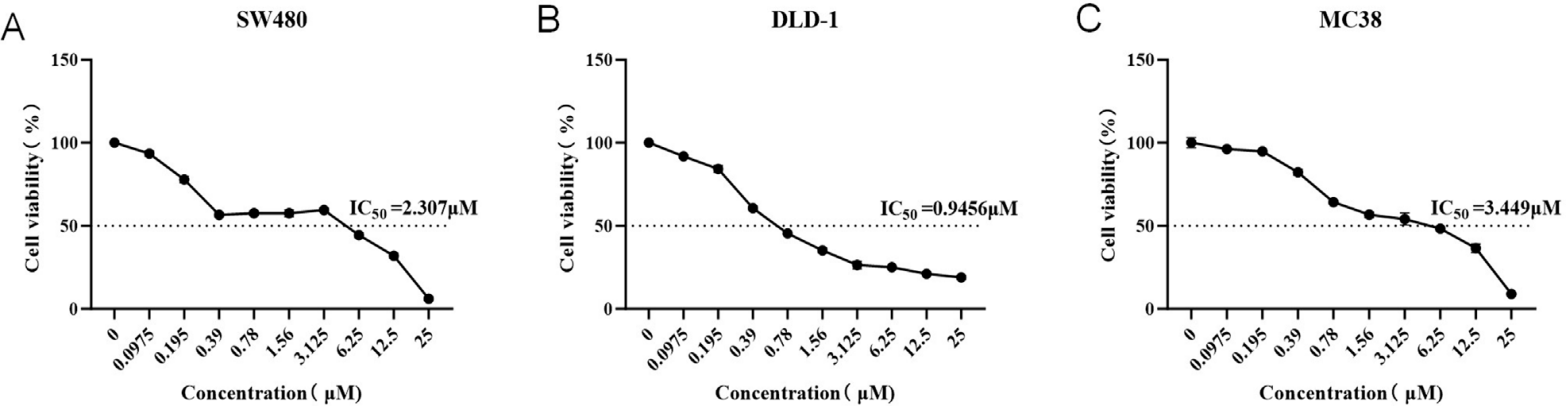
MTT assay viability assay for three CRC cell lines. **(A)** SW-480; **(B)** DLD-1; **(C)** MC38.

The MTT assay was used to assess the effects of SB-225002 on CRC cell viability (SW-480 cells, DLD-1 cells and MC 38 cells). Results showed a dose-dependent inhibition of cell viability. The IC_50_ value of SB-225002 toward SW-480 cells was 2.307 μM. The IC_50_ value of SB-225002 toward DLD-1 cells was 0.9456 μM. The IC_50_ value of SB-225002 toward MC 38 cells was 3.449 μM, indicating that SB-225002 significantly inhibited the proliferation of CRC cells at lower concentrations (Figure 12).

## 4 Discussion

CRC is currently the third most common cancer worldwide, accounting for approximately 10% of all cancer cases. It is also the second leading cause of cancer-related mortality globally. The primary treatment strategy for CRC remains surgical resection, supplemented by chemotherapy and radiotherapy. However, the efficacy of chemotherapy has reached a plateau, and molecular targeted therapy has become the main approach to improving the prognosis of patients with advanced CRC. The molecular mechanisms underlying CRC initiation and progression remain to be fully elucidated, making it essential to identify novel biomarkers with clinical, pathological, and prognostic significance to guide CRC treatment (Lişcu et al., 2024). Targeted therapy, as an emerging approach, enhances the precision of chemotherapy by selectively targeting cancer cells while preserving normal tissues (Svec et al., 2024). Given the current limitations in predictive biomarkers for CRC, the early prediction and targeted treatment of CRC, as well as the identification of effective and specific biomarkers, remain critical tasks.

In this study, A total of 15 DEGs with *p* < 0.05, including *MELK*, *NFE2L3*, *MCM2*, *MAD2L1*, *AUNIP*, *CXCL3*, *GLDN*, *GREM2*, *ALDH1A1*, *CILP, FABP4*, *AOC3*, *CNN1*, *ANGPTL1*, and *DES*, were identified for subsequent drug screening research. Some of these genes (such as *CXCL3* (Liao et al., 2025), *MCM2* (Tian et al., 2024), *NFE2L3* (Saliba et al., 2022), *MAD2L1* (Gharebaghi et al., 2024), *MELK* (Tang et al., 2022), *ANGPTL1* (Chang et al., 2022), *GREM2* (Zhang and Zhu, 2022), *GLDN* (Su et al., 2022), *VAP-1* (Li et al., 2014), *FABP-4* (Zhou et al., 2014), *ALDH1A1* (Wang X. et al., 2025), *CNN1* (Wang L. et al., 2025), *DES* (Ma et al., 2009)) have previously been confirmed to be associated with CRC, which further validates our research results. However, the roles of *AUNIP* and *CILP* in this malignant tumor have not been fully studied. Detailed information can be found in Tables 3 and 4. In the future, our research will focus on these differentially expressed genes to elucidate their specific contributions to the tumorigenesis of CRC.

**TABLE 3:** Detailed information on the six upregulated genes in CRC research.

Gene symbol	Names	Impact on CRC
*AUNIP*	Aurora Kinase A and Ninein Interacting Protein	There are no relevant studies on AUNIP in the field of CRC. In a study on oral squamous cell carcinoma, it was found that AUNIP regulates the cell cycle, and knocking down AUNIP leads to G0/G1 phase arrest in OSCC cells (Yang et al., 2019)
*CXCL3*	C-X-C Motif Chemokine 3	CXCL3 can be activated by the JAK2/STAT3 pathway and promotes the progression of CRC by reducing CD8^+^ T cells (Liao et al., 2025)
*MCM2*	Minichromosome maintenance complex component 2	The USP31-RUNX1 pathway exerts regulatory effects on MCM2. USP31 stabilizes RUNX1 through deubiquitination, thereby enhancing its transcriptional activity. RUNX1 directly binds to the regulatory region of MCM2, promoting its expression. Overexpression of MCM2 accelerates DNA replication, cell cycle progression, and proliferation in colon cancer cells, and is associated with poor prognosis (Tian et al., 2024)
*NFE2L3*	Nuclear factor erythroid 2-related factor 3	NFE2L3 upregulates IL33 and inhibits RAB27A, thereby promoting mast cell activation sand the release of inflammatory factors, creating a pro-cancer inflammatory microenvironment; it can also directly regulate the cell cycle to promote the proliferation of CRC cells (Saliba et al., 2022)
*MAD2L1*	Mitotic arrest deficient-2 like-1	MAD2L1 is a tumor suppressor gene that can regulate the cell cycle and maintain the normal order of cell growth and division. In CRC, the downregulation of miR - 6787–5p (a microRNA that targets and controls MAD2L1) affects the expression of MAD2L1, thereby promoting the proliferation of cancer cells and the progression of CRC (Gharebaghi et al., 2024)
*MELK*	Maternal embryonic leucine zipper kinase	The expression of MELK is regulated by xCT, which promotes CRC development by upregulating the oncogene MELK and activating the AKT/mTOR cascade (Tang et al., 2022)

**TABLE 4:** Detailed information on the nine downregulated genes in CRC research.

Gene symbol	Names	Impact on CRC
*ANGPTL1*	Angiopoietin-like protein 1	ANGPTL1 inhibits the expression of SRY-related HMG-box (SOX)-2 (SOX2) by enhancing the expression of forkhead box O (FOXO) -3a (FOXO3a), thereby suppressing the development of CRC (Chang et al., 2022)
*GREM2*	Gremlin-2	MiR-103a-3p drives CRC development through the regulation of GREM2 (Zhang and Zhu, 2022)
*GLDN*	Gliomedin	Previous studies have linked GLDN to hepatocellular carcinoma (Zhang et al., 2020) and gastric cancer (You et al., 2024; Liu et al., 2024). In CRC, Ying Su et al. identified GLDN as a prognostic biomarker for colon cancer based on machine learning and bioinformatics analysis (Su et al., 2022)
*VAP-1*	Vascular Adhesion Protein-1	Research on VAP-1 in CRC is limited. Yu-I Li et al. reported that VAP-1 can predict all-cause mortality and cancer-specific mortality in patients with CRC (Li et al., 2014)
*FABP-4*	Fatty Acid-Binding Protein 4	FABP-4 primarily participates in the transport of fatty acids to cellular compartments, the regulation of intracellular lipid metabolism, and the modulation of gene expression (Boord et al., 2002). FABP-4 may influence the development of CRC through its effects on inflammation (Zhou et al., 2014) and insulin resistance (Pisani, 2008; Jenab et al., 2007; Rinaldi et al., 2008; Murphy et al., 2022)
*ALDH1A1*	Aldehyde Dehydrogenase 1 Family Member A1	ALDH1A1 facilitates CRC metastasis by activating the Notch signaling pathway (Wang et al., 2025a)
*CILP*	Cartilage Intermediate Layer Protein	CILP is an extracellular matrix protein that is abundant in cartilage tissue. It is primarily associated with musculoskeletal disorders, including osteoarthritis and lumbar disc degeneration (Mori et al., 2006), and most current studies focus on its role in connective tissue. At present, research on CILP in CRC is nearly nonexistent; however, Ye Jin Ha et al. identified CILP2 as a potential biomarker for predicting and targeting peritoneal metastasis in CRC (Ha et al., 2024)
*CNN1*	Calponin 1	Ultrasound-targeted microbubble destruction (UTMD)-mediated upregulation of CNN1 induces ferroptosis in CRC cells by regulating p53-related SLC7A11 expression, highlighting CNN1 as a potential target for therapeutic intervention and diagnostic strategies in CRC (Wang et al., 2025b)
*DES*	Desmin	DES encodes a muscle-specific type III intermediate filament. Currently, many studies have investigated Desmin in CRC, and evidence has confirmed that Desmin is a potential oncofetal diagnostic and prognostic biomarker for CRC (Ma et al., 2009)

L1000FWD, DGIdb and CMap platforms were used to screen drugs, and molecular docking analysis was performed. Although some genes identified here (e.g., *CXCL3*, *MCM2, MELK*) are known in CRC (Liao et al., 2025; Tian et al., 2024; Tang et al., 2022), our study distinguishes itself through a rigorous integrative bioinformatics workflow focused on druggable target discovery. We prioritized not only differential expression but also prognostic significance and validation in a clinical cohort, yielding a small but robust set of high-confidence genes. Drug prediction analysis applied to this refined gene set has uncovered potential therapeutic agents, such as SB-225002, highlighting the pipeline’s utility for translational research.

Further *in vitro* experiments verified SB-225002s efficacy and safety, establishing it as an ideal therapeutic agent for CRC, but the direct evidence regarding the anti-inflammatory and anti-metastatic effects of SB-225002 in CRC remains limited. However, as a selective CXCR2 antagonist (White et al., 1998), SB-225002 has demonstrated promising therapeutic activity across multiple other cancer types through diverse mechanisms. For example, in breast cancer, SB-225002 was shown to suppress bone metastasis by inhibiting the CXCL5–CXCR2 signaling axis (Romero-Moreno et al., 2019). In nasopharyngeal carcinoma, it exerted anti-tumor effects by reducing phosphorylated AKT (p-AKT) levels (Lo et al., 2013). Similarly, in prostate cancer, SB-225002 inhibited AKT phosphorylation, thereby blocking the PI3K signaling pathway, while also suppressing the secretion of bone sialoprotein and osteopontin, ultimately attenuating metastatic progression (Xu et al., 2018). In lung cancer, it reduced neutrophil infiltration in the tumor microenvironment and enhanced CD8^+^ T lymphocyte activation, contributing to anti-tumor immunity (Cheng et al., 2021). Furthermore, in mantle cell lymphoma, SB-225002 overcame drug resistance by inhibiting the activation of Akt, STAT3, and p38 signaling pathways and reprogramming lymphoma-associated macrophages (Sun et al., 2025). Given these broad mechanisms observed in other malignancies, our future research will focus on elucidating the anti-inflammatory and anti-metastatic potential of SB-225002 specifically in the context of CRC.

## 5 Conclusion

Through the analysis of multiple TCGA datasets, *ACSL6, AUNIP, CXCL3, CXCL8, CXCL11, EPHX4, GPR143, LRRC8, MAD2L1, MCM2, MELK, MMP3,* and *NFE2L3* were identified as potential therapeutic biomarkers for CRC. Internal and external database validation further confirmed the predictive significance of 15 key genes. Among them, SB-225002 emerged as a promising candidate for CRC treatment. Subsequent *in vitro* experiments validated the efficacy and safety of SB-225002, making it a potential therapeutic agent for CRC. However, further research is required to evaluate the clinical potential of SB-225002 as a targeted therapy for CRC.

## Data Availability

The datasets generated in this study have been deposited in the National Population Health Data Center Data Warehouse (PHDA, 2024) under the DOI https://doi.org/10.12213/11.Z0I2Q.202411.267.V1.0, and all other datasets used in the analyses are available from the corresponding public repositories as described in the manuscript.

## Glossary

ALDH1A1: Aldehyde Dehydrogenase 1 Family Member A1
ANGPTL1: Angiopoietin-like protein 1
ANOVA: analysis of variance
AOC3: amine oxidase copper containing 3
AUNIP: Aurora kinase A and ninein-interacting protein
BMPs: bone morphogenetic proteins
BP: biological process
CC: cellular component
CILP: Cartilage Intermediate Layer Protein
CMap: Connectivity Map
CNN1: Calponin 1
CRC: colorectal cancer
CXCL3: C-X-C Motif Chemokine 3
DEGs: differentially expressed genes
DES: Desmin
DGIdb: Drug-Gene Interaction Database
DMSO: dimethyl sulfoxide
FABP-4: Fatty Acid-Binding Protein 4
FC: fold change
FPKM: Fragments Per Kilobase of transcript per Million mapped reads
GEO: Gene Expression Omnibus
GLDN: Gliomedin
GO: Gene Ontology
GREM2: Gremlin-2
HPA: Human Protein Atlas
KEGG: Kyoto Encyclopedia of Genes and Genomes
L1000FWD: L1000 Fireworks Display
MAD2L1: Mitotic arrest deficient-2 like-1
MCM2: Minichromosome maintenance complex component 2
MELK: Maternal embryonic leucine zipper kinase
MF: molecular function
MOA: mechanisms of action
NFE2L3: Nuclear factor erythroid 2-related factor 3
OD: optical density
OS: overall survival
PCA: Principal Component Analysis
PRDC: Protein Related to DAN and Cerberus
SD: standard deviation
TCGA: The Cancer Genome Atlas
TGF-β: transforming growth factor-beta
UniProt: Universal Protein
VAP-1: Vascular Adhesion Protein-1
